# Serum activin A and B levels predict outcome in patients with acute respiratory failure: a prospective cohort study

**DOI:** 10.1186/cc13093

**Published:** 2013-10-31

**Authors:** David Morritz de Kretser, Jonathan Guy Bensley, Ville Pettilä, Rita Linko, Mark Peter Hedger, Susan Hayward, Carolyn Anne Allan, Robert Ian McLachlan, Helen Ludlow, David James Phillips

**Affiliations:** 1 Monash Institute of Medical Research, Monash University, PO Box 5418, Clayton, Victoria 3168, Australia; 2 Department of Anatomy and Developmental Biology, Monash University, Clayton, Victoria 3800, Australia; 3 Intensive Care Unit, Department of Anesthesia and Intensive Care Medicine, Division of Surgery, Helsinki University Hospital, Helsinki, Finland; 4 Prince Henry’s Institute, Clayton, Victoria 3168, Australia; 5 Oxford Brookes University, Oxford, UK

## Abstract

**Introduction:**

30 day mortality in patients with Acute Respiratory Failure (ARF) is approximately 30%, defined as patients requiring ventilator support for more than 6 hours. Novel biomarkers are needed to predict patient outcomes and to guide potential future therapies. The activins A and B, members of the Transforming Growth Factor β family of proteins, and their binding protein, follistatin, have recently been shown to be important regulators of inflammation and fibrosis but no substantial data are available concerning their roles in ARF.

Our objectives were to evaluate whether the serum levels of activin A, B and follistatin are elevated in 518 patients with ARF from the FINNALI study compared the concentrations in 138 normal subjects that form a reference range.

**Methods:**

Specific assays for activin A, B and follistatin were used and the results analyzed according to diagnostic groups as well as according to standard measures in intensive care. Multivariable logistic regression was used to create a model to predict death at 90 days and 12 months from the onset of the ARF.

**Results:**

Serum activin A and B were significantly elevated in most patients and in most of the diagnostic groups. Patients who had activin A and/or B concentrations above the reference maximum were significantly more likely to die in the 12 months following admission [either activin A or B above reference maximum: Positive Likelihood Ratio [LR+] 1.65 [95% CI 1.28-2.12, *P* = 0.00013]; both activin A and B above reference maximum: LR + 2.78 [95% CI 1.96-3.95, *P* < 0.00001]. The predictive model at 12 months had an overall accuracy of 80.2% [95% CI 76.6-83.3%].

**Conclusions:**

The measurement of activin A and B levels in these patients with ARF would have assisted in predicting those at greatest risk of death. Given the existing data from animal studies linking high activin A levels to significant inflammatory challenges, the results from this study suggest that approaches to modulate activin A and B bioactivity should be explored as potential therapeutic agents.

## Introduction

Managing acute respiratory failure (ARF) in the ICU is a major challenge [1-5]. Mortality in ARF, defined as requiring the need for ventilation for more than 6 hours, is approximately 30% at 30 days after ARF onset [3,6-8], and despite many advances in care, improved methods are required to define those at greatest risk and to predict outcomes. Identifying novel biomarkers in ARF may lead to potential new therapies and enable better prediction of short and long term outcomes.

Activin A, a dimer of βA subunits, is a member of the transforming growth factor-β [TGFβ] superfamily, and was isolated originally for its capacity to stimulate follicle-stimulating hormone secretion by the pituitary gland. The amino acid sequence of the βA subunit is 100% conserved from the mouse to the human [9]. In experimental animal models in sheep and mice, it was subsequently shown to be a major stimulator of the inflammatory cascade initiated by lipopolysaccharide (LPS) [10,11] and drives inflammation and fibrosis in various pathologies [9]. Another member of this family is activin B, a dimer of βB subunits, with 70% amino acid sequence homology to βA [9]. These proteins are produced in multiple organs and tissues [9].

Many factors regulate activin A bioactivity but follistatin is regarded as the major regulator, binding activin A virtually irreversibly and targeting the complex to a lysosomal degradation pathway [12,13]. Activin A stimulates follistatin production, thereby modulating its own biological actions [9,11] and a high activin A to follistatin ratio favors pro-inflammatory and fibrotic processes that are decreased by follistatin [14]. Administration of follistatin to mice before they were given LPS, resulted in a markedly lower TNFα response and altered the magnitude and temporal secretory pattern of IL1β and IL6 [15]. Further, follistatin administration prior to a lethal LPS injection was found to halve the mortality in mice [15].

A recent study used the intra-tracheal administration of an adenoviral associated vector expressing activin A in mice and showed that it induced a profound inflammatory response resulting in a cytokine storm and the transformation of normal lungs to an emphysematous phenotype in 3 to 4 weeks in the surviving mice [16].

Activin B, a closely related member of the TGFβ-activin protein subfamily, binds to the same receptor and is regarded as a weak activin A agonist, but there are very limited data concerning its actions in inflammation [9]. Its bioactivity can also be blocked by follistatin.

There are only limited data about the role of activins A and B in humans. A small study conducted in critically ill patients with septicemia suggested that markedly elevated serum activin A levels were associated with an increased risk of death [17]. In part, the absence of well-characterized assays for activins A and B and follistatin, and normal-range values obtained from a substantial number of volunteers carefully selected to exclude illnesses, particularly those with an inflammatory component, has hindered the acquisition of human data. Such well-defined normal ranges are critical to using serum activin A, B and follistatin as sensitive markers of inflammation. Previously defined normal ranges for activin A are inadequate due to limited numbers of normal subjects [18,19].

This study establishes normal reference ranges for serum activin A and B and follistatin levels, and reports their levels in a large cohort of critically ill patients with ARF in the FINNALI study [3]. This study also establishes that serum activin A and B levels may assist in predicting survival outcomes of patients with ARF and provides a rationale to explore therapeutic avenues to modulate activin A and B.

## Materials and methods

### Ethics

The Board of the Finnish Quality Consortium (Intensium Ltd, Kuopio, Finland) and the Ethics Committees of each participating hospital approved the study design and use of the quality database for the purposes of the FINNALI study [3]. Due to the established nature of the ICU care provided in each ICU, the ethics committees waived the requirement for informed consent for data registration. The normal-range study was approved by the Southern Health Human Ethics Committee, Clayton, Victoria, Australia.

### Samples from the FINNALI Study

**Figure 1:**
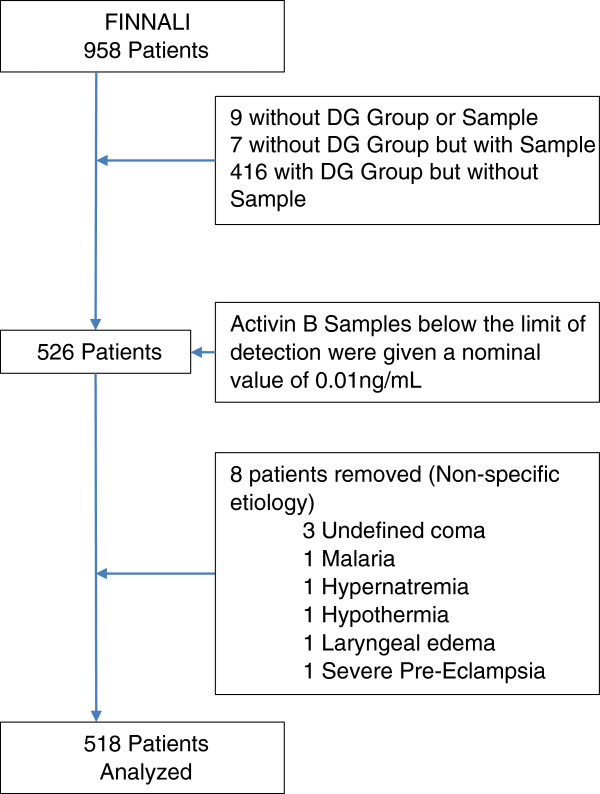
**Patient selection flowchart.** The initial selection step was the presence of an Acute Physiology and Chronic Health Evaluation (Apache) III diagnostic group and a sample available for analysis. Activin B samples below the limit of detection were given a nominal value to enable further analysis. Patients with non-specific etiologies were removed to enable examination of specific etiologies in this study.

From the FINNALI study of 958 patients with ARF treated by any ventilator support (invasive or non-invasive) for more than 6 hours and sequentially admitted to the ICU [3], 518 patients were selected solely on the basis of available blood samples (Figure 1). Their clinical management followed standard practice in the 12 participating ICUs. Blood samples were taken within 6 hours of commencing ventilation on day (D) zero (D0) (n = 487) and on D2 (n = 495), D7 (n = 323) and D21 (n = 62). Of 518 patients, only 47 had all four samples drawn. This analysis, unless otherwise noted, utilized the D0, D2 and D7 samples.

Acute lung injury (ALI) or Acute Respiratory Distress Syndrome (ARDS) was diagnosed using the American-European Consensus Conference criteria [20]. Mortality was calculated from the beginning of the ARF. Of the 518 patients included in this study, 17 patients (3.3% of patients) had ARDS and 27 patients (5.2% of patients) had ALI at the beginning of ARF. The remainder, 474 patients, did not have ALI or ARDS at the beginning of ARF.

### Patient data

Patient data were grouped by APACHE III diagnostic group [3]. Diagnostic groups are outlined in Table 1, and patient selection is detailed in Figure 1. The presence of chronic morbidities and risk factors preceding ARF, Simplified Acute Physiology Score (SAPS) II [21] minus oxygen points, day 1 Sequential Organ Failure Assessment (SOFA) score [22] minus respiration points and the APACHE III score [23] were recorded. Outcome measures were mortality at 90 days and 12 months.

**Table 1:** Diagnostic categories of patients

**Group**	**Diagnosis**	**Number of patients**
	**Postoperative patients**	
1	Cardiovascular	118
2	Gastrointestinal	57
	Non-operative patients	
3	Cardiovascular	74
4	Sepsis	26
5	Gastro-intestinal/hematologic	24
6	Metabolic	22
7	Neurologic	56
8	Respiratory	84
9	Trauma	37
	Postoperative miscellaneous	
10	Respiratory/gynecology/renal/orthopedic	20

Patients grouped on the basis of their diagnostic categories as defined according to Acute Physiology and Chronic Health Evaluation (APACHE III) diagnostic groups (3).

### Normal range study

Healthy adult male and female patients (n = 138) in the following age groups were recruited: 18 to 50 years (n = 56), 51 to 65 years (n = 52) and over 66 years (n = 30). Eligibility required body mass index (BMI) of <30, no major hospitalization within the last 3 years, no current acute illness or injury, no chronic inflammatory or neoplastic disease and no medication for significant clinical disease.

Following interview by the nurse coordinator, volunteers were formally consented by the study coordinator (DJP) and completed a form outlining any pertinent medical details. The following data were stored and used for statistical analyses: sex, age, ethnicity, weight, height, BMI, hip and waist circumference, details of hospitalizations, allergies, tobacco and alcohol usage (approximate number of standard alcoholic drinks (10 g alcohol or equivalent] per week), details of any medications and, if taking cholesterol-lowering medication, they were excluded from the study. Details of exercise (hours per week of exercise beyond normal lifestyle activity), symptoms potentially indicative of health problems (either past or present) were recorded including the date of last menstrual period (for premenopausal women). A sample of 120 mL of blood was taken from each volunteer, allowed to clot and the serum from each sample was aliquoted into smaller individual volumes and stored at -20°C to enable normal ranges to be determined for new assays.

### Immunoassays

Total serum activin A was measured by a two-site ELISA [24] with a sensitivity of 7.7 pg/mL and intra- and inter-assay coefficients of variation of 5.7% and 7.7%, respectively. Total serum activin B was measured by ELISA as previously described [25] with no cross-reactivity with inhibin B or activin A. The assay sensitivity is 0.019 ng/mL and the intra- and inter-assay coefficients of variation range between 2.7 to 6.2%, and 5.5 to 11.7%, respectively. Total follistatin was measured by radioimmunoassay as previously published [26] with a sensitivity of 1.44 ng/mL and intra- and inter-assay coefficients of variation of 5.8% and 7.1%, respectively.

### Statistical analysis

SPSS 21 (IBM, New York, NY USA), SPSS Modeler 14.2 (IBM), SAS 9.3 (SAS, New York, NY USA) and GraphPad Prism 6 (GraphPad, La Jolla, Ca USA) were used to analyze data. Analyses used were the Kruskal-Wallis test with Dunn’s multiple comparison post-hoc test (all multiple comparison analyses), Mann-Whitney *U*-Test (normal range comparisons) and the Chi-squared test (for age survival analysis). The predictive model was created using a binary logistic regression. Validation was performed using bootstrapping [27,28]. Hosmer-Lemeshow tests, area under curve (AUC), positive likelihood ratio (LR) with 95% CIs, accuracy with 95% CIs, net reclassification index (NRI) and the integrated discrimination improvement index (IDI) were calculated [29]. Survival evaluation used a log-rank test. Data are reported as mean ± standard error of the mean (SEM) and 95% CI, with a *P*-value less than 0.05 considered statistically significant. Exact statistics are reported.

For the purposes of the predictive model, although substantially more accurate models were possible, they were unlikely to be generalizable, contained too many terms or would be difficult to perform at multiple time points. The threshold was set at 0.5, as we would regard a false positive and a false negative as equally bad outcomes.

## Results

### Normal range study

#### Activin A

**Figure 2:**
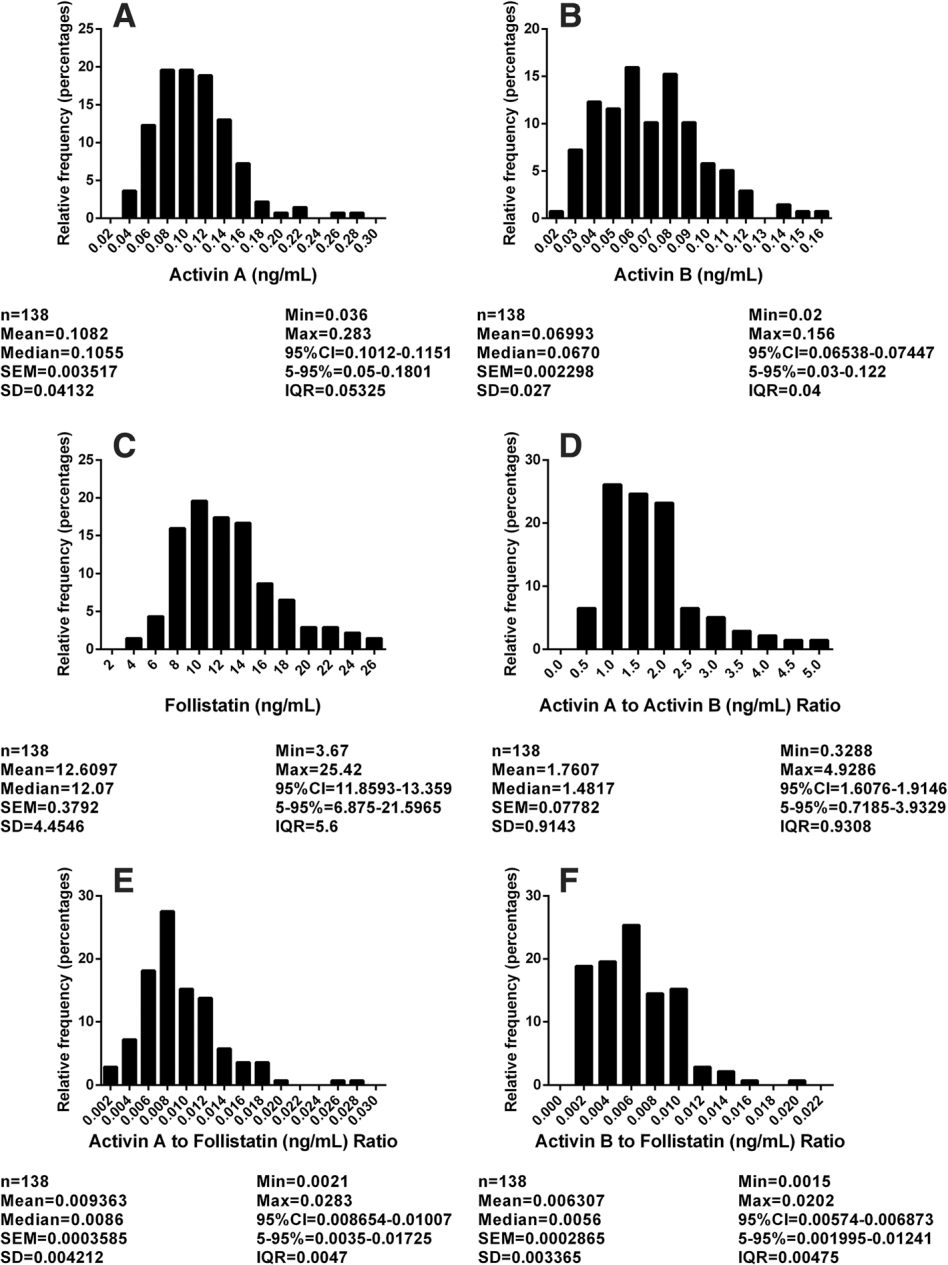
**Reference ranges from normal subjects, depicted as (A) activin A (ng/mL), (B) activin B (ng/mL), (C) follistatin (ng/mL), (D) activin A to activin B ratio, (E) activin A to follistatin ratio, and (F) activin B to follistatin ratio.** In each panel, n = number of subjects. SEM, standard error of the mean; Min, minimum; Max, maximum; 95% CI, 95% confidence interval; 5-95%, 5 to 95% values.

Serum Activin A concentrations were 0.11 ± 0.41 ng/mL (range 0.036 to 0.283 ng/mL) and significantly increased with age, regardless of gender (*r*^2^ = 0.25, *P* <0.0001). Multivariate analysis showed a significant contribution of ethnicity (*P* = 0.011). Although potential participants were excluded if they were taking cholesterol-lowering medications, a proportion of participants were taking other medications. There were no significant effects of weight, hip circumference, alcohol consumption, smoking, time since hospitalization, exercise, allergies, or days since last menstruation. There were minor correlations with height (*r*^2^ = 0.031, *P* = 0.039), BMI (*r*^2^ = 0.028, *P* = 0.048) and waist circumference (*r*^2^ = 0.03, *P* = 0.042; Additional file 1: Figure E1). Activin A concentrations for each age group of 18 to 50 years, 51 to 65 years and 66+ years are shown in Table 2 and Figure 2.

**Table 2:** Normal ranges for serum activin A, B and follistatin

**Analyte**	**Sex**	**Age (years)**	**Number**	**Concentration (ng/mL)**	**95% CI**
Activin A	M/F	18 to 50	56	0.087 ± 0.004	0.080, 0.095
		51 to 65	52	0.110 ± 0.005	0.099, 0.120
		66+	30	0.150 ± 0.009	0.130, 0.170
Activin B	F	18 to 50	29	0.085 ± 0.005	0.074, 0.096
		51 to 65	31	0.062 ± 0.005	0.051, 0.073
		66+	20	0.071 ± 0.005	0.060, 0.082
Activin B	M	18 to 50	27	0.061 ± 0.004	0.053, 0.069
		51 to 65	21	0.064 ± 0.005	0.053, 0.074
		66+	10	0.086 ± 0.008	0.069, 0.100
Follistatin	M/F	18 to 50	56	11.67 ± 0.63	10.40, 12.94
		51 to 65	52	12.61 ± 0.58	11.44, 13.78
		66+	30	14.36 ± 0.71	12.90, 15.82

Reference ranges for activin A, activin B and follistatin, presented as mean ± standard error of the mean concentrations and 95% CI M, male; F, female.

#### Activin B

Serum activin B concentrations were 0.070 ± 0.002 ng/mL (range 0.020 to 0.156 ng/mL). There were no significant differences with ethnicity and hypertensive medications. Age and sex were significantly correlated with activin B levels (*P* = 0.002). In female patients, activin B concentrations decreased with age, whereas in male patients they increased with age. Data for male and female patients appear separately in Table 2, and additional data are shown in Figure 2. These data have been published previously as part of the validation of the activin B assay [25] but are included in this paper to assist the reader in evaluating the use of this parameter in the management of patients with ARF.

#### Follistatin

Serum follistatin concentrations were 12.61 ± 0.38 ng/mL (range 3.67 to 25.42 ng/mL). There were no significant correlations with sex, ethnicity, waist or hip circumference, or BMI. Serum follistatin levels correlated with the number of days since the last menstrual period (*r*^2^ = 0.29, *P* = 0.004). Follistatin concentrations were positively correlated with age (*P* = 0.004). Data for each age range are shown in Table 2 and Figure 2.

### Association of activins A and B, and follistatin, and survival at 12 months

This study used samples from a subset of patients from the FINNALI study [3]. The subset of patients included in this study were significantly less likely to die, stayed about a day longer in ICU and were less likely to be an emergency admission compared to the original FINNALI study (Additional file 1: Table E1).

#### Activin A levels

**Figure 3:**
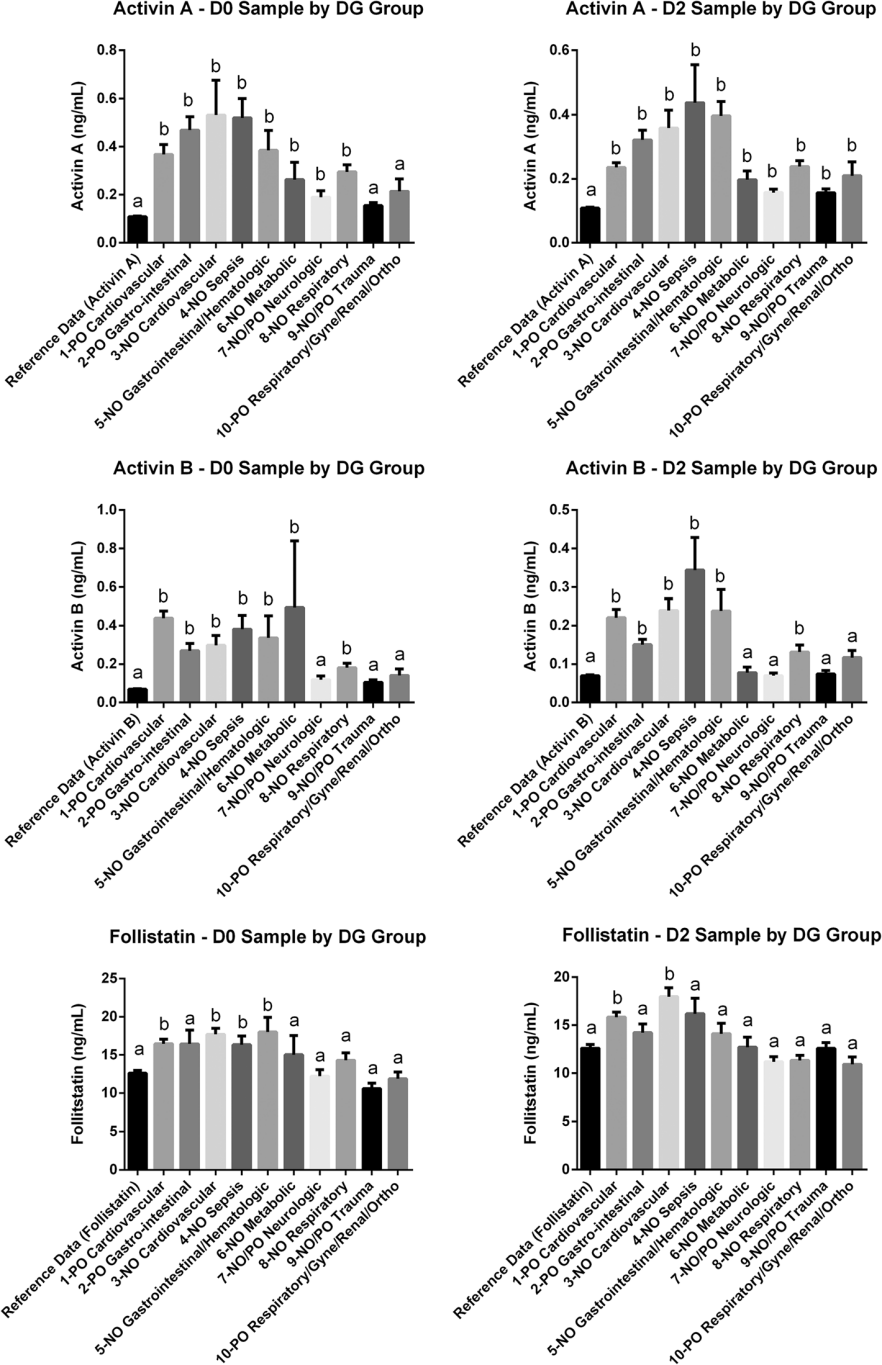
**Activin A/B and follistatin concentrations in ARF patients at day zero (D0) or day 2 (D2), compared with reference group levels (reference data).** Each patient group was sorted by diagnosis (DG Group). Differing letters (a or b) denote a significant difference from the reference data. (NO) non-operative and (PO) Post-operative.

Sample D0 (within 6 hours from the commencement of ventilation) activin A levels did not differ between male and female patients and were markedly elevated (*P* <0.0001) above the normal range in all diagnostic groups (Figure 3, Additional file 1: Table E6) other than in the non-operative/postoperative trauma (group 9) and the postoperative respiratory, gynecology, renal and orthopedic groups (group 10).

Serum activin A levels measured at D0 in patients who died at 90 days and 12 months were also markedly elevated above normal (*P* <0.0001). There were no significant differences between patients who survived and those who had died at those time points (Additional file 1: Table E1). In contrast, at the D2 time point (two days after the commencement of ventilation) serum activin A levels were significantly different between those who were alive and those who died at 90 days (*P* = 0.008) and 12 months (*P* = 0.0003; Additional file 1: Table E2). However, at the D7 sample time point, there were no differences. The rate of change between samples D0, D2 and D7 did not differ between those who lived and those who had died.

#### Activin B levels

In sample D0 serum activin B concentrations did not differ significantly between the sexes and were markedly elevated above the normal range in groups 1 to 6 and group 8 (groups 1 to 5, and 8, *P* <0.0001; group 6, *P* = 0.025) but were not significantly elevated in non-operative/postoperative neurologic (group 7), non-operative/postoperative trauma (Group 9), and the postoperative respiratory, gynecology, renal and orthopedic group (group 10, Figure 3 and Additional file 1: Table E6). In D0 samples, activin B levels were elevated (*P* <0.0001) above reference levels, but did not differ between those who survived and who had died at 90 days, or at 12 months (Additional file 1: Table E2). For both the D2 and D7 samples, serum activin B levels were significantly higher in those who died at 90 days and 12 months compared with those surviving at those times (Additional file 1: Table E2).

#### Follistatin levels

In the D0 sample, follistatin levels did not differ between the sexes and were elevated significantly above the normal range in groups 1 (*P* <0.0001), 3 (*P* <0.0001), 4 (*P* = 0.024), and 5 (*P* = 0.008) (Figure 3 and Additional file 1: Table E6). Using sample D0, patients had significantly (*P* = 0.0004) higher follistatin levels compared to the normal range, but there were no differences between those who were dead or alive at 90 days, or at 12 months (Additional file 1: Table E2). In the D2 samples, serum follistatin levels were higher than the reference subjects in patients dying at 90 days (*P* = 0.0007) and 12 months (*P* = 0.0018). Among those surviving to 90 days and 12 months, serum follistatin levels were lower than the normal range cohort (*P* = 0.008 and *P* = 0.033, respectively) for the D7 time point only. Serum follistatin levels in samples D2 and D7 samples were higher in those who had died at 90 days and at 12 months compared with those who lived, except for death at 12 months in the D2 sample (Additional file 1: Table E2).

#### ALI/ARDS

We also evaluated the effect of having ALI or ARDS on the levels of activins and follistatin. Of the 518 patients included in this study, we had data on ALI/ARDS for 495 patients. Of those 495 patients, 27 had ALI and 17 had ARDS. Patients with ALI did have statistically significantly higher levels of serum activin A at time points D2 (*P* = 0.04) and D7 (*P* = 0.01), but not at D0, when compared to patients without ALI. There were no differences in activin B or follistatin levels for patients with ALI compared with patients without ALI. When comparing patients with ALI who were alive and those who died at 90 days and 12 months, no differences in serum activins and follistatin levels were found at any time point.

Comparing ARDS patients with patients without ARDS, no differences were detected in serum activins or follistatin at any time point examined. Comparing ARDS patients who lived and died at 90 days and 12 months, no differences in serum activins and follistatin levels were found at any time point.

#### Survival

Gender did not affect survival at any time point. Age significantly increased the probability of death at 90 days and 12 months, using logistic regression (90 days (odds ratio (OR) per year 1.034, 95% CI 1.019, 1.049; *P* <0.00001), 12 months (OR per year 1.034, 95% CI 1.021, 1.048; *P* <0.00001). When evaluating survival using three groups, patients aged 16 to 50, 51 to 65 and 65+ years, patients who were over 65 years of age had 2.6 times greater risk of death at 12 months compared with patients who were 16 to 50 years of age (*P* = 0.0002), and patients aged 65+ had 1.87 times greater risk of death than those aged 51 to 65 years old (*P* = 0.0067), but there was no difference between those aged 16 to 50 and those aged 51 to 65 years ( *P* = 0.11). At 90 days, patients who were aged over 65 years had 3.1 times greater risk of death compared to patients aged 16 to 50 years (*P* <0.0001), patients aged 65+ had 1.9 times greater risk of death than those aged 51 to 65 years old (*P* = 0.0043), there was no effect between those aged 16 to 50 and those aged 51 to 65 years ( *P* = 0.26).

### Predictive model

In order to formulate a scoring system that would facilitate the use of measures of the activins and follistatin by clinicians, we assessed a variety of models to determine their value in predicting patient outcome. We utilized MDLP (minimal description length principle) optimal scaling, maximum, mean and upper/lower 95% CI of the normal range to determine if activin A/B and follistatin levels would provide useful adjuncts to existing scoring systems in predicting survival.

For D0 samples, concentrations above the reference maximum, or any other metric for activin A, activin B and follistatin, were not useful in predicting outcome at 90 days or at 12 months. For D2 Samples, activin A and B offered good performance in predicting outcome at 90 days and 12 months, using the reference maximum as the cutoff point (Additional file 1: Table E3). Follistatin did not offer useful predictions using any metric. Although D7 samples offered good predictive value, we elected not to use them for modeling. A sample drawn at 7 days after the commencement of ventilation is not a practical time point for clinical use. Further, the use of multiple activins/follistatin measurements at multiple time points was not considered practical.

**Figure 4:**
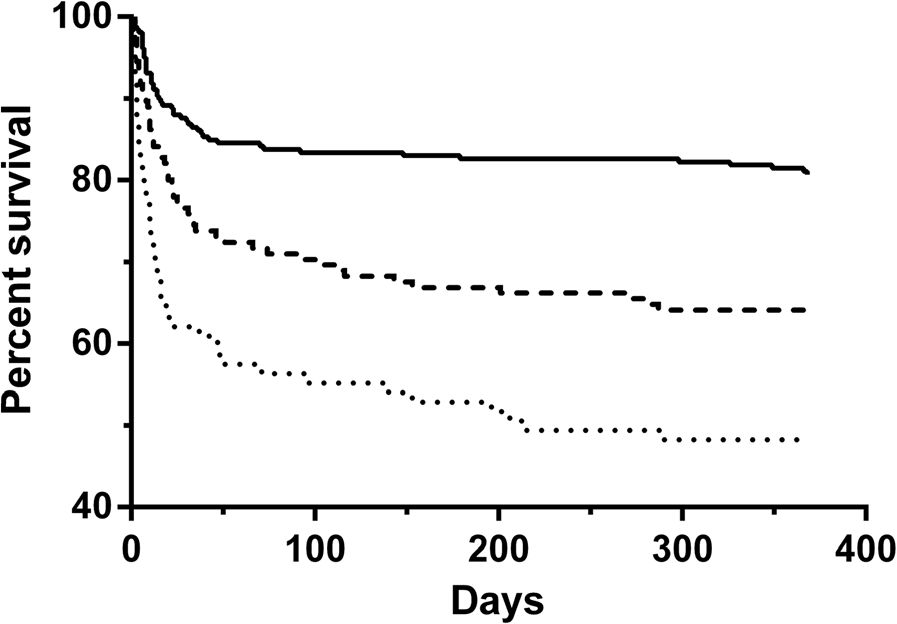
**Patient survival curves of up to 12 months according to activins A/B in the day 2 (D2) sample.** Solid line: when neither activin A or activin B were over the reference maximum; dashed line: when either activin A or activin B were over the reference maximum (dashed line); dotted line: when both activin A and activin B were over the reference maximum. Statistically, comparing neither activin A or B over the reference maximum to either activin A or B over the reference maximum yielded *P* = 0.0003; comparing neither activin A or B over the reference maximum to both activin A or B over the reference maximum yielded *P* <0.0001, and comparing either activin A or B over the reference maximum to both activin A and B over over the reference maximum yielded *P* = 0.009.

Based on these results, we used sample D2 for predicting ICU survival. We therefore used a constructed variable for activin A and B at time-point D2 with a three-option design, to evaluate specifically: when neither activin A or B were above the reference maximum; when either activin A or B were above the reference maximum, or when both activin A and B were above the reference maximum. This approach gave a good separation for survival curves, and was a good starting point for modeling (Figure 4). These constructed variables were robust using the AUC, positive LR, relative risk and observed risk measures (Additional file 1: Table E4).

We then developed a scoring system, the follistatin, and activin A and B (FAB) score to predict the risk of death over the year after the initiation of ventilation. Details of the components incorporated into the score are given in the legend to Table 3; as well as measures of activins A, and B, the score utilizes several standard criteria routinely collected in the management of critically ill patients in the ICU. This is a balanced score, calibrated at both 90 days (Hosmer-Lemeshow test, Chi-square = 9.502, *P* = 0.302) and 12 months (Chi- square = 12.222, *P* = 0.142).

**Table 3:** Components of the FAB prediction model

**Variable**	**Type**		**Equation factor**
Age	Numeric (Years)		0.041
Activin A, Activin B measurement at 2 days after commencement of ventilation – FAB Constructed variable	Choice	Activin A <0.283 ng/mL	0
		Activin B <0.156 ng/mL	
		Activin A >0.283 ng/mL OR	0.934
		Activin B >0.156 ng/mL	
		Activin A >0.283 ng/mL AND	1.197
		Activin B >0.156 ng/mL	
Chronic restrictive pulmonary disease	Yes/No	Yes	0.900
Chronic obstructive pulmonary disease	Yes/No	Yes	0.577
Diagnostic group	Choice	Postoperative cardiovascular	0
		Postoperative gastrointestinal	1.505
		Non-operative cardiovascular	2.135
		Non-operative sepsis	0.959
		Non-operative Gastrointestinal/hematological	1.111
		Non-operative metabolic	1.796
		Non-operative neurologic	2.599
		Non-operative respiratory	1.824
		Non-operative trauma	0.720
		Miscellaneous postoperative (respiratory, gynecologic, renal, orthopedic)	1.265
SOFA maximal score	Numeric		0.220
Constant			-7.765

FAB, follistatin, and activin A and B; SOFA: Sequential Organ Failure Assessment. Formula for FAB score: (Age*0.041) + (Activin A/B component) + (Presence of xhronic restrictive pulmonary disease) + (Presence of chronic obstructive pulmonary disease) + (Diagnostic group) + (Maximal SOFA score*0.220) + Constant. This produces a range with a mean of zero. To obtain the risk profile, each value was entered into the following equation:

$$\text{Probability}\;\text{Estimate}=\frac{\ln\text{Result}}{1+\ln\text{Result}}$$ .

This produced a range of values from 0.0 to 1.0, a score approaching 1.0 having the greatest risk of death and approaching 0.0 having the least risk of death. For risk stratification purposes, it was then divided into 10 bins, each 0.1 wide with the upper endpoint of each bin included in the lower bin.

The equation factor listed is the value that should be inserted into the equation.

Comparing the FAB score without the activin A/B component with the complete FAB score demonstrated that the addition of the activin A/B component substantially improved the predictive value of the model for outcomes at both 90 days and 12 months.

For 90 days, the addition of the activin A/B component yielded a categorical NRI of 0.0058 (95% CI -0.0565, 0.0682; *P* = 0.854), a category-free NRI of 0.4444 (95% CI 0.2375, 0.6513; *P* = 0.00001), and an IDI of 0.0268 (95% CI 0.008, 0.0457; *P* = 0.0053). For 12 months, the addition of the activin A/B component yielded a categorical NRI of 0.0414 (95% CI: -0.0205, 0.1032; *P* = 0.190), a category-free NRI of 0.4942 (95% CI 0.2997, 0.6887; *P* <0.000001) and an IDI of 0.0322 (95% CI 0.015, 0.0495; *P* = 0.00025).

**Figure 5:**
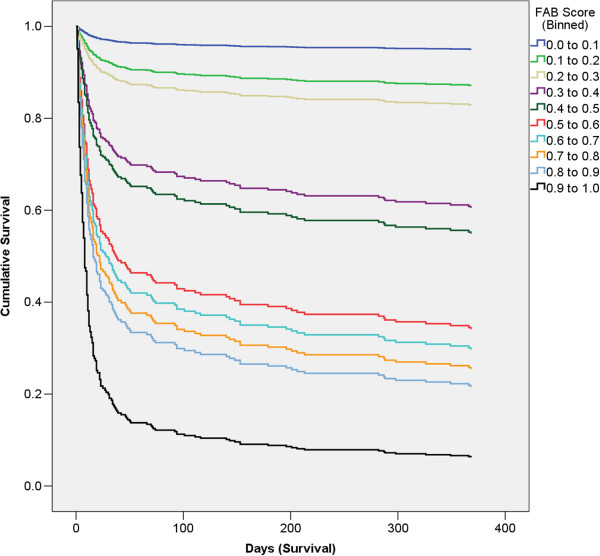
**Survival curves by FAB score (binned).** FAB Scores closer to 0.0 have the lowest risk of death, and those closest to 1.0 have the greatest risk of death. FAB, follistatin, and activin A and B.

To better stratify risk, the FAB score for purposes of this analysis was divided from 0.0 to 1.0 in 10 equal bins (each 0.1 wide), with the top value included in the lower of each bin (Figure 5). The ORs and other details are shown in Additional file 1: Table E5.

### Use of this predictive model in the individual diagnostic groups

The use of this predictive model in the individual diagnostic groups was not equally predictive in each group. As is evident in Figure 3, not all the diagnostic groups had levels of activin A and B above the reference mean or reference maximum and hence in our predictive model the diagnostic group is used to account for differences in the relative mortality risk in each group. Using the FAB score and applying it to each diagnostic group individually demonstrates that it performed well, statistically significantly above the null hypothesis area of 0.5 for on the receiver operating characteristic (ROC) curve, in all but three of the groups (Additional file 1: Table E7). This is primarily due to the lower numbers in groups 4, 9 and 10. Another factor is that the outcomes can be remote from the causative factor for the ARF and that some groups are heterogeneous.

## Discussion

This paper provides a unique dataset on serum levels of activin A and B, and follistatin in critically ill patients, had ARF, and were in the ICU. These data, with the establishment of normal ranges for the assays based on substantive numbers of healthy volunteers, enable a critical appraisal of the value of these measurements for diagnostic and predictive purposes. The findings of this study establish that the concentrations of both activin A and B are substantially increased in most of the diagnostic groups of critically ill patients with ARF. This is in keeping with the demonstration that activin A is a key regulator of the inflammatory response induced by LPS [11] and the small study that indicated that there were substantial increases in patients in the ICU with septicemia [9].

In addition, the present study demonstrates that serum activin B levels were substantially elevated in a large proportion of this critically ill cohort. Further, having activin B levels above the reference maximum (combined with other factors) gives the best predictor of mortality subsequent to admission. This finding is of interest, given that although clear data in mice and humans indicate that activin A is a major controller of the inflammatory response [9,11,30], very little is known about the role of activin B in inflammation and fibrosis.

The advantages of measuring activin A and B are that an elevated measurement of these proteins (A and/or B) at 2 days after the initiation of ventilation, still has predictive power at 12 months after admission to the ICU. Adding the activin A and B measurements improves upon the already robust base model (Table 3), as shown by the significant NRI and IDI results, and therefore identifies patients who are currently not detected as being of higher mortality risk.

Activin A stimulates macrophages to produce nitric oxide [31], and increasing activin A levels results in apoptosis of hepatocytes and B lymphocytes, resulting in liver failure and suppression of immunological responses, both of which can cause death [32,33]. Further, activin A stimulates fibroblast mitosis *in vitro* and in vivo, as well as expression of metallo-matrix proteases in macrophages after experimental induction of myocardial infarction [34-36]. The latter is consistent with the elevated activin A levels in group 3 consisting of patients with non-operative cardiovascular conditions associated with ARF, and with Ynestad and colleagues [36], who noted that the magnitude of the increase in activin A levels paralleled the increasing severity of the cardiac failure. In this group, activin B levels were also elevated, again providing a novel finding, the pathophysiological basis of which is unknown. Apart from the capacity for activin B as well as activin A to cause apoptosis of MPC-11 cells, there is a paucity of data concerning its actions [37] other than experiments wherein substitution of the βB subunit for βA in mice suggested that the βB subunit acted as a partial agonist when compared to the βA subunit [38].

This large patient cohort adds substantially to the available data in humans and further supports the concept that activin A, and now activin B, are valuable markers of inflammatory responses. We recognize that this paper provides data from a study performed some time ago. A new prospective study is now required that builds in the analysis of the activins and follistatin in the management of patients with ARF in the ICU.

The importance of activin A as a major regulator of the inflammatory response is supported by its capacity to stimulate monocytes/macrophages to produce a number of inflammatory mediators including IL1β, TNFα, IL6, nitric oxide, prostaglandin E2 and thromboxane [39-41]. Further, studies in mouse models and human data clearly establish a role for this protein in inflammatory bowel disease, rheumatoid arthritis, allergy-induced asthma and wound healing [14,42-45]. Further, studies following the progress of patients with multiple forms of inflammatory diseases using the activins and follistatin as markers would establish the value of using the levels of both activins A and B as markers of organ function in inflammation.

**Figure 6:**
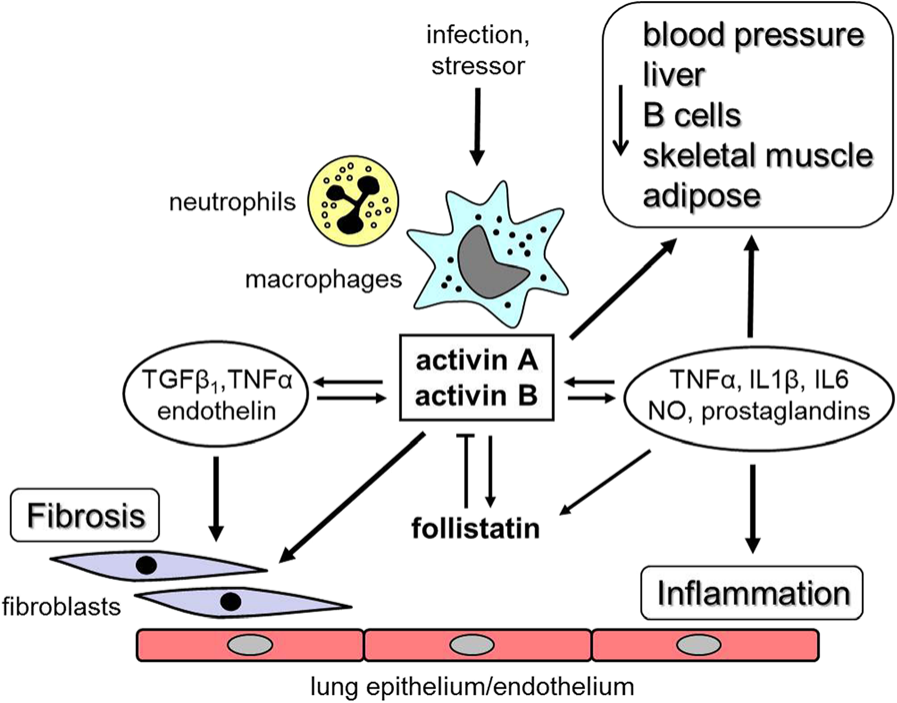
**Diagrammatic summary of the actions of the activins as they pertain to the lung.** These proteins are produced in neutrophils, macrophages, fibroblasts, the endothelium and the bronchial epithelium, as well as multiple sites in other tissues, details of which are found in the review by Hedger *et al*. [9]. (NO) Nitric oxide.

There may be multiple factors linking these proteins to survival and these are summarized in Figure 6. In acute experiments that used a lethal LPS challenge in mice, those with the highest activin A levels had the greatest mortality [11]. Proof that these elevated activin A levels caused death emerged from halving the LPS-induced mortality by the administration of follistatin, which binds activin A virtually irreversibly and targets the complex to a lysosomal degradation pathway [12,13,46]. Although not formally addressed, the rapid action of follistatin in halving the LPS-induced mortality, usually within 24 hours, may result from blocking the rapid increase in nitric oxide arising from the stimulation of macrophages by activin A, causing hypotension [31]. However, the longer-term impact on survival and its prediction by the measurement of activins A and B are likely to be related to the capacity of elevated levels of activin A to cause apoptosis of hepatocytes, decreasing liver function and compromising immunological defense mechanisms resulting from the apoptosis of B lymphocytes. In addition, elevated activin A levels drive fibrosis and this may lead to limitation of pulmonary, hepatic and renal function [9,34,41,44]. Further, elevated activin A levels can cause cachexia resulting from activin A signaling through type II and type I receptors [47]. This pathway, shared by myostatin, leads to the stimulation of the Smad2/3/4 pathway resulting in the dephosphorylation of the transcription factor foxo3a and leading to its nuclear retention [48]. The increased levels of nuclear foxo3a induce the expression of muscle specific ubiquitin ligases, MuRF-1 and Atrogin-1, which stimulate the degradation of myofibrillar proteins such as myosin, resulting in marked muscle wasting [49]. Although a role for activin B in cachexia is yet to be established, activin B can act through the same signaling pathway via Smad 2/3/4 and also can activate the pathway via ALK7, another type 1 receptor [50].

Defining the pathways through which high activin A levels cause death is of critical importance as therapeutic options will become available using follistatin, which has the capacity to bind and neutralize the actions of both activin A and B. Further, the development of soluble activin receptor blockers offer alternative possibilities for more selective treatment as follistatin also inhibits the actions of several of the bone morphogenetic proteins, growth and differentiation factor (GDF) 8 (myostatin), GDF9, and TGFβ3 [9].

In this context, it was surprising that the activin to follistatin ratio did not correlate with long-term survival and it was clear that concentrations of follistatin were higher in those patients who died than those who survived, at D7. In part, this probably results from the increased levels of activin A and B, measured in these severely ill patients, to stimulate follistatin levels.

## Conclusions

This study establishes that activins A and B, novel modulators of inflammation and fibrosis, are elevated in a large number of patients with ARF in the ICU. It also demonstrates the value of measuring their levels in the management of critically ill patients in intensive care, as patients with elevated levels of both activins above the normal range are more likely to die at 3 and 12 months after their admission to the ICU with ARF. These higher mortality rates are the likely result of the ability of increased levels of activin A to cause inflammation and the production of nitric oxide, and induce apoptosis of hepatocytes and B lymphocytes. Further, as activin A also induces fibrosis in multiple tissues, such as the lung, liver and kidney, it has the capacity to cause sub-optimal organ function, thereby contributing to poor survival [8,11,41]. These data should stimulate the development of inhibitors of the action of the activins such as follistatin as therapeutic agents.

## Key messages

• ARF, particularly in the ICU, results in significant mortality. Novel biomarkers are needed to predict patient outcomes and guide potential future therapies.

• We demonstrated that the novel modulators of inflammation and fibrosis, activins A, B and follistatin, are elevated in the majority of patients with ARF and marked increases are linked to poor outcomes.

• The severity of the increases in serum levels of activins A and B can assist in the prediction of the outcomes in ARF and helps to identify those patients at greatest risk of death, thereby establishing the basis for the development of new therapeutic agents targeted at modulating the bioactivity of these protein biomarkers.

## Abbreviations

ALI: Acute lung injury; APACHE: Acute physiology and chronic health evaluation; ARDS: Acute respiratory distress syndrome; ARF: Acute respiratory failure; AUC: Area under the curve; BMI: Body mass index; ELISA: Enzyme linked immunosorbent assay; FAB score: Follisatin, Activin A and B score; GDF8: growth differentiation factor 8 (myostatin); GDF9: Growth differentiation factor 9; IDI: Integrated discrimination improvement index; IL: Interleukin; LPS: Lipopolysaccharide; LR: Likelihood ratio; MDLP: Minimum description length principle; NRI: Net reclassification index; OR: Odds ratio; ROC: Receiver operating characteristic; SAPS: Simplified acute physiology score; SEM: Standard error of the mean; SOFA: Sequential organ failure assessment; TGFβ: Transforming growth factor beta; TNFα: Tumor necrosis factor alpha.

## Competing interests

DdeK is a director of Paranta Biosciences, a company developing follistatin as a therapeutic and, together with DJP, holds shares in the company recognizing their intellectual input into the provisional patents, licensed to Paranta. JGB, VP, RL, MPH, SH, CA, RIMcL, and HL declare no conflict of interest. Supported by a donation from Mrs Margaret Ross and a grant from Diagnostic Systems Laboratories (now a Beckman Coulter Company).

## Authors’ contributions

Project conception, design, analysis and interpretation: all authors. Data acquisition and interpretation: DdeK, JGB, VP, RL, MH, SH, CA, RMcL, DJP. Development of the activin B assay: HL. Drafting of article: DdeK, JGB, DJP. Critical revision of article: all authors. Final article approval: all authors.

## Supplementary Material

Additional file 1: Figure E1 Correlation of serum concentrations of activin A (ng/mL) in controls according to (**A**) height (centimeters), (**B**) body mass index (BMI), and (**C**) waist circumference (centimeters). **Table E1.** Details of the patients in the current study compared to the compete cohort in the original FINNALI study. **Table E2.** Details of serum activin A and B, and follistatin in patients at day (D)0, D2 and D7. **Table E3.** Ratios of serum activin A and B to the maximum normal reference value for each analyte. **Table E4.** Data for the follisatin, and activin A and B (FAB)-constructed variable. **Table E5.** Detailed stratification of FAB scores. **Table E6.** Data for diagnostic group at each time point (D0, D2 and D7) for follistatin, and activin A and B. **Table E7.** receiver operating characteristic (ROC) analysis for the FAB score at 90 days and 12 months, organized by individual diagnostic group. Click here for file
