# Supplementary material for: Serum activin A and B levels predict outcome in patients with acute respiratory failure: a prospective cohort study
Source: Crit Care. 2013 Oct 31;17(5):R263. doi: 10.1186/cc13093 (PMC4057391; doi:10.1186/cc13093)
Supplement: Additional file 1: Figure E1 — Correlation of serum concentrations of activin A (ng/mL) in controls according to (A) height (centimeters), (B) body mass index (BMI), and (C) waist circumference (centimeters). Table E1. Details of the patients in the current study compared to the compete cohort in the original FINNALI study. Table E2. Details of serum activin A and B, and follistatin in patients at day (D)0, D2 and D7. Table E3. Ratios of serum activin A and B to the maximum normal reference value for each analyte. Table E4. Data for the follisatin, and activin A and B (FAB)-constructed variable. Table E5. Detailed stratification of FAB scores. Table E6. Data for diagnostic group at each time point (D0, D2 and D7) for follistatin, and activin A and B. Table E7. receiver operating characteristic (ROC) analysis for the FAB score at 90 days and 12 months, organized by individual diagnostic group. [file cc13093-S1.docx]

**Additional Data**

**Normal Range Study:**

Supplementary Figure E1: Correlation of serum concentrations of activin A (ng/mL) in controls according to (A) height (centimeters), (B) BMI, and (C) waist circumference (centimeters).

Supplementary Table E1: Details of the patients in the current study compared to the compete cohort in the original FINNALI study.

Supplementary Table E2: Details of Serum Activin A, B and Follistatin in Patients at D0, D2 and D7

Supplementary Table E3: Ratios of Serum activin A and B to the maximum normal reference value for each analyte.

Supplementary Table E4: Data for the FAB constructed variable.

Supplementary Table E5: Detail Stratification of FAB Scores

Supplementary Table E6: Data for Diagnostic Group at each time point (D0, D2 and D7) for Follistatin, Activin A and Activin B

Supplementary Table E7: ROC analysis for the FAB Score at 90 days and 12 months, organised by individual diagnostic group

Supplementary Figure E1


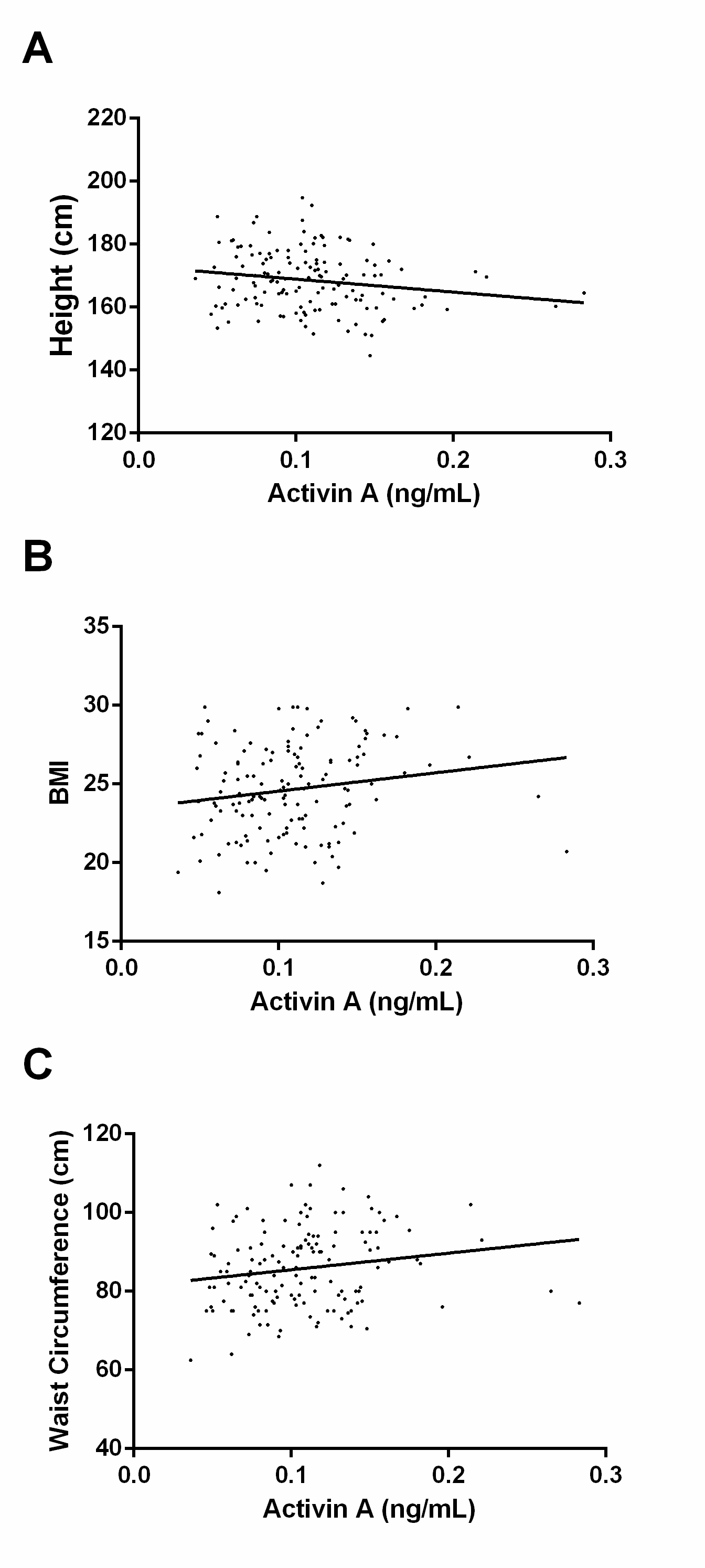


| **Parameter** | **FINNALI (958 patients)** | | | | | | | **This Study (518 patients)** | | | | | | | ***P*-value** |
| --- | --- | --- | --- | --- | --- | --- | --- | --- | --- | --- | --- | --- | --- | --- | --- |
|  | n | n(t) | % | Mean | SEM | Min-Max | 95% CI | n | n(t) | % | Mean | SEM | Min-Max | 95% CI |  |
| Male | 637 | 958 | 66.5% | - | - | - | 0.64-0.70 | 357 | 518 | 68.9% | - | - | - | 0.65-0.73 | 0.34 |
| Female | 321 | 958 | 33.5% | - | - | - | 0.31-0.37 | 161 | 518 | 31.1% | - | - | - | 0.27-0.35 | 0.34 |
| Age | 958 | 958 | 100.0% | 60.37 | 0.55 | 16-94 | 59.29-61.44 | 518 | 518 | 100.0% | 61.11 | 0.75 | 16-90 | 59.64-62.57 | 0.42 |
| Weight (kilograms) | 427 | 958 | 44.6% | 81.52 | 0.93 | 36-164 | 76.69-83.34 | 354 | 518 | 68.3% | 81.54 | 1.044 | 36-164 | 79.49-83.59 | 0.99 |
| Height (centimeters) | 425 | 958 | 44.4% | 172.18 | 0.44 | 147-193 | 171.31-173.04 | 352 | 518 | 68.0% | 172.4 | 0.49 | 147-193 | 171.45-173.36 | 0.73 |
| BMI | 941 | 958 | 98.2% | 26.99 | 0.19 | 14-66.6 | 26.61-27.36 | 515 | 518 | 99.4% | 27.28 | 0.27 | 14-66.6 | 26.75-27.80 | 0.37 |
| Apache Score | 958 | 958 | 100.0% | 23.51 | 0.28 | 3-57 | 22.95-24.06 | 518 | 518 | 100.0% | 22.67 | 0.36 | 6-49 | 21.96-23.37 | 0.072 |
| SAPS Score | 958 | 958 | 100.0% | 44.05 | 0.56 | 2-117 | 42.95-45.15 | 518 | 518 | 100.0% | 42.12 | 0.72 | 2-94 | 40.70-43.53 | *0.037 |
| SOFA Score (24hrs after admission) | 958 | 958 | 100.0% | 8.00 | 0.11 | 0-18 | 7.78-8.22 | 518 | 518 | 100.0% | 7.66 | 0.14 | 0-18 | 7.39-7.93 | 0.068 |
| ICU LOS (Days) | 958 | 958 | 100.0% | 5.57 | 0.23 | 0.17-74.13 | 5.13-6.02 | 518 | 518 | 100.0% | 6.517 | 0.35 | 0.4-74.13 | 5.82-7.21 | *0.019 |
| Hospital LOS (Days) | 958 | 958 | 100.0% | 16.88 | 0.61 | 0-205 | 15.68-18.08 | 518 | 518 | 100.0% | 18.67 | 0.79 | 0-187 | 17.12-20.22 | 0.078 |
| Died in ICU | 120 | 958 | 12.5% | - | - | - | 0.10-0.15 | 38 | 518 | 7.3% | - | - | - | 0.051-0.096 | *0.0021 |
| Died in Hospital | 230 | 958 | 24.0% | - | - | - | 0.21-0.27 | 96 | 518 | 18.5% | - | - | - | 0.15-0.22 | *0.016 |
| Died ≤ 30 Days | 251 | 958 | 26.2% | - | - | - | 0.23-0.29 | 104 | 518 | 20.1% | - | - | - | 0.17-0.24 | *0.0086 |
| Died ≤ 90 Days | 295 | 956 | 30.9% | - | - | - | 0.28-0.34 | 129 | 518 | 24.9% | - | - | - | 0.21-0.29 | *0.017 |
| Died ≤ 6 Months | 318 | 958 | 33.2% | - | - | - | 0.30-0.36 | 141 | 518 | 27.2% | - | - | - | 0.23-0.31 | *0.018 |
| Died ≤ 12 Months | 338 | 958 | 35.3% | - | - | - | 0.32-0.38 | 156 | 518 | 30.1% | - | - | - | 0.26-0.34 | *0.045 |
|  |  |  |  |  |  |  |  |  |  |  |  |  |  |  |  |
| Chronic Heart Disease | 371 | 942 | 39.4% | - | - | - | 0.36-0.43 | 212 | 512 | 41.4% | - | - | - | 0.37-0.46 | 0.45 |
| Chronic Obstructive Lung Disease | 158 | 938 | 16.8% | - | - | - | 0.14-0.19 | 94 | 510 | 18.4% | - | - | - | 0.15-0.22 | 0.45 |
| Chronic Restrictive Lung Disease | 36 | 934 | 3.9% | - | - | - | 0.026-0.051 | 24 | 510 | 4.7% | - | - | - | 0.029-0.065 | 0.44 |
| Current Smoker | 259 | 797 | 32.5% | - | - | - | 0.29-0.36 | 137 | 460 | 29.8% | - | - | - | 0.26-0.34 | 0.32 |
| Diabetic | 179 | 940 | 19.0% | - | - | - | 0.17-0.22 | 99 | 514 | 19.3% | - | - | - | 0.16-0.23 | 0.92 |
| Emergency Admission | 821 | 954 | 86.1% | - | - | - | 0.84-0.88 | 422 | 515 | 81.9% | - | - | - | 0.79-0.85 | *0.037 |
| Infection | 178 | 562 | 31.7% | - | - | - | 0.28-0.36 | 146 | 431 | 33.9% | - | - | - | 0.29-0.38 | 0.46 |
| Intubation Tube | 818 | 953 | 85.8% | - | - | - | 0.84-0.88 | 440 | 515 | 85.4% | - | - | - | 0.82-0.89 | 0.84 |
| NYHA Class 1 | 621 | 933 | 66.6% | - | - | - | 0.64-0.70 | 312 | 505 | 61.8% | - | - | - | 0.58-0.66 | 0.07 |
| NYHA Class 2 | 159 | 933 | 17.0% | - | - | - | 0.15-0.20 | 92 | 505 | 18.2% | - | - | - | 0.15-0.22 | 0.58 |
| NYHA Class 3 | 83 | 933 | 8.9% | - | - | - | 0.07-0.11 | 59 | 505 | 11.7% | - | - | - | 0.089-0.15 | 0.091 |
| NYHA Class 4 | 70 | 933 | 7.5% | - | - | - | 0.058-0.092 | 42 | 505 | 8.3% | - | - | - | 0.059-0.11 | 0.58 |
| Obesity (BMI>35) | 76 | 941 | 8.1% | - | - | - | 0.063-0.098 | 45 | 512 | 8.8% | - | - | - | 0.063-0.11 | 0.64 |
| Operative | 375 | 958 | 39.1% | - | - | - | 0.36-0.42 | 220 | 518 | 42.5% | - | - | - | 0.38-0.47 | 0.22 |
| Renal Replacement Therapy | 97 | 471 | 20.6% | - | - | - | 0.17-0.24 | 59 | 367 | 16.1% | - | - | - | 0.12-0.20 | 0.094 |
|  |  |  |  |  |  |  |  |  |  |  |  |  |  |  |  |
| 1-PO Cardiovascular | 176 | 958 | 18.4% | - | - | - | - | 118 | 518 | 22.8% | - | - | - | - | - |
| 2-PO Gastro-intestinal | 98 | 958 | 10.2% | - | - | - | - | 57 | 518 | 11.0% | - | - | - | - | - |
| 3-NO Cardiovascular | 142 | 958 | 14.8% | - | - | - | - | 74 | 518 | 14.3% | - | - | - | - | - |
| 4-NO Sepsis | 47 | 958 | 4.9% | - | - | - | - | 26 | 518 | 5.0% | - | - | - | - | - |
| 5-NO Gastrointestinal/Hematologic | 40 | 958 | 4.2% | - | - | - | - | 24 | 518 | 4.6% | - | - | - | - | - |
| 6-NO Metabolic | 53 | 958 | 5.5% | - | - | - | - | 22 | 518 | 4.2% | - | - | - | - | - |
| 7-NO/PO Neurologic | 115 | 958 | 12.0% | - | - | - | - | 56 | 518 | 10.8% | - | - | - | - | - |
| 8-NO Respiratory | 129 | 958 | 13.5% | - | - | - | - | 84 | 518 | 16.2% | - | - | - | - | - |
| 9-NO/PO Trauma | 72 | 958 | 7.5% | - | - | - | - | 37 | 518 | 7.1% | - | - | - | - | - |
| 10-PO Respiratory/Gyne/Renal/Ortho | 42 | 958 | 4.4% | - | - | - | - | 20 | 518 | 3.9% | - | - | - | - | - |
| X-NO Other medical disease | 28 | 958 | 2.9% | - | - | - | - | - | - | - | - | - | - | - | - |
| Missing | 16 | 958 | 1.7% | - | - | - | - | - | - | - | - | - | - | - | - |

Supplementary Table E1: Details of the patients in the current study compared to the compete cohort in the original FINNALI study.

Comparison of various parameters in the FINNALI study (3) with the equivalent parameter in the current study. n indicates the number of subjects in which that variable was present, n(t) indicates the number of subjects for which a record was available, for each variable. The *P*-values compare the equivalent parameter in the FINNALI study with the current study; *=*P*<0.05

Supplementary Table E2: Details of Serum Activin A, B and Follistatin in Patients at D0, D2 and D7

Mean ± SEM, Median and Interquartile Range (IQR) for concentrations of activin A, activin B and follistatin at time-points D0, D2 and D7. For each analyte and time point, patients were divided into those who were alive or died at 90 days and 12 months. At each point, statistical comparisons were made of those who were alive or dead and with the relevant reference range: d*a compares died vs. alive, d*r compared died vs. reference range and a*r compared alive vs. reference range. d=dead, a=alive and r=reference value; **P*< 0.05

| Analyte |  |  |  | 90 Days | 12 Months |
| --- | --- | --- | --- | --- | --- |
| Activin A | Sample D0 | Dead | Mean±SEM | 0.39±0.034 (n=129) | 0.41±0.04 (n=156) |
|  |  |  | Median/IQR | 0.26/0.32 | 0.27/0.31 |
|  |  | Alive | Mean±SEM | 0.34±0.033 (n=358) | 0.33±0.033 (n=331) |
|  |  |  | Median/IQR | 0.21/0.22 | 0.21/0.33 |
|  |  |  |  | d*a=0.0921  d*r=<0.0001*  a*r=<0.0001* | d*a=0.0580  d*r=<0.0001*  a*r=<0.0001* |
|  | Sample D2 | Dead | Mean±SEM | 0.30±0.02 (n=123) | 0.33±0.027 (n=150) |
|  |  |  | Median/IQR | 0.22/0.26 | 0.23/0.26 |
|  |  | Alive | Mean±SEM | 0.25±0.015 (n=372) | 0.24±0.013 (n=345) |
|  |  |  | Median/IQR | 0.19/0.16 | 0.18/0.14 |
|  |  |  |  | d*a=0.0082*  d*r=<0.0001*  a*r=<0.0001* | d*a=0.0003*  d*r=<0.0001*  a*r=<0.0001* |
|  | Sample D7 | Dead | Mean±SEM | 0.31±0.024 (n=65) | 0.31±0.026 (n=79) |
|  |  |  | Median/IQR | 0.26/0.26 | 0.25/0.25 |
|  |  | Alive | Mean±SEM | 0.29±0.025 (n=257) | 0.28±0.026 (n=243) |
|  |  |  | Median/IQR | 0.21/0.16 | 0.2/0.17 |
|  |  |  |  | d*a=0.4703  d*r=<0.0001*  a*r=<0.0001* | d*a=0.2913  d*r=<0.0001*  a*r=<0.0001* |
| Activin B | Sample D0 | Dead | Mean±SEM | 0.34±0.062 (n=129) | 0.32±0.052 (n=156) |
|  |  |  | Median/IQR | 0.17/0.17 | 0.17/0.17 |
|  |  | Alive | Mean±SEM | 0.26±0.018 (n=358) | 0.27±0.019 (n=331) |
|  |  |  | Median/IQR | 0.15/0.24 | 0.15/0.24 |
|  |  |  |  | d*a=0.1618  d*r=<0.0001*  a*r=<0.0001* | d*a=0.2532  d*r=<0.0001*  a*r=<0.0001* |
|  | Sample D2 | Dead | Mean±SEM | 0.23±0.026 (n=123) | 0.23±0.023 (n=150) |
|  |  |  | Median/IQR | 0.14/0.19 | 0.14/0.16 |
|  |  | Alive | Mean±SEM | 0.15±0.0093 (n=372) | 0.15±0.0090 (n=345) |
|  |  |  | Median/IQR | 0.10/0.11 | 0.10/0.12 |
|  |  |  |  | d*a=0.0018*  d*r=<0.0001*  a*r=<0.0001* | d*a=0.0008*  d*r=<0.0001*  a*r=<0.0001* |
|  | Sample D7 | Dead | Mean±SEM | 0.22±0.037 (n=65) | 0.20±0.03 (n=80) |
|  |  |  | Median/IQR | 0.14/0.17 | 0.13/0.14 |
|  |  | Alive | Mean±SEM | 0.13±0.0087 (n=258) | 0.13±0.009 (n=243) |
|  |  |  | Median/IQR | 0.09/0.09 | 0.092/0.09 |
|  |  |  |  | d*a=0.0001*  d*r=<0.0001*  a*r=<0.0001* | d*a=0.0001*  d*r=<0.0001*  a*r=<0.0001* |
| Follistatin | Sample D0 | Dead | Mean±SEM | 15.86±0.71 (n=129) | 15.80±0.62 (n=156) |
|  |  |  | Median/IQR | 14.32/7.41 | 14.29/7.29 |
|  |  | Alive | Mean±SEM | 15.07±0.44 (n=358) | 15.03±0.47 (n=331) |
|  |  |  | Median/IQR | 13.69/7.95 | 13.62/7.96 |
|  |  |  |  | d*a=0.9406  d*r=0.0011*  a*r=0.0026* | d*a=0.5958  d*r=0.0004*  a*r=0.0046* |
|  | Sample D2 | Dead | Mean±SEM | 15.25±0.54 (n=123) | 15.16±0.53 (n=150) |
|  |  |  | Median/IQR | 14.02/8.14 | 13.76/8.02 |
|  |  | Alive | Mean±SEM | 13.76±0.32 (n=372) | 13.68±0.32 (n=345) |
|  |  |  | Median/IQR | 13.03/6.61 | 13.21/6.71 |
|  |  |  |  | d*a=0.0207*  d*r=0.0007*  a*r=0.2334 | d*a=0.0581  d*r=0.0018*  a*r=0.2401 |
|  | Sample D7 | Dead | Mean±SEM | 13.7±0.58 (n=65) | 13.37±0.52 (n=80) |
|  |  |  | Median/IQR | 13.86/5.57 | 13.5/5.85 |
|  |  | Alive | Mean±SEM | 11.43±0.33 (n=258) | 11.39±0.35 (n=243) |
|  |  |  | Median/IQR | 10.5/5.94 | 10.45/5.91 |
|  |  |  |  | d*a=<0.0001*  d*r=0.2180  a*r=0.0096* | d*a=0.0002*  d*r=0.5160  a*r=0.0078* |

Supplementary Table E3: Ratios of Serum activin A and B to the maximum normal reference value for each analyte.

Data for activin A and B over the reference maximum value, measured at the D2 time-point, for death at 90 days and 12 months. This includes the area under curve (AUC), the positive Likelihood Ratio (LR+), the Relative Risk (RR) and the Odds Ratio (OR).

| Activin A - D2 Sample - Over Reference Maximum |  | **AUC** | **95% CI** | ***P* value** | **LR+** | ***P* value** | **95% CI** | **RR** | **95% CI** | **OR** | **95% CI** |
| --- | --- | --- | --- | --- | --- | --- | --- | --- | --- | --- | --- |
|  | 90 days | 0.57 | 0.51-0.63 | 0.02 | 1.54 | 0.004 | 1.17-2.04 | 1.60 | 1.06-1.44 | 1.90 | 1.24-2.93 |
|  | 12 months | 0.59 | 0.53-0.64 | 0.0022 | 1.72 | 0.00013 | 1.31-2.24 | 1.70 | 1.31-2.21 | 2.22 | 1.48-3.35 |
| Activin B - D2 Sample - Over Reference Maximum |  | **AUC** | **95% CI** | ***P* value** | **LR+** | ***P* value** |  | **RR** | **95% CI** | **OR** | **95% CI** |
|  | 90 days | 0.58 | 0.53-0.64 | 0.005 | 1.57 | 0.00078 | 1.22-2.01 | 1.70 | 1.26-2.29 | 2.06 | 1.35-3.13 |
|  | 12 months | 0.57 | 0.52-0.63 | 0.009 | 1.50 | 0.0016 | 1.18-1.92 | 1.54 | 1.19-2.01 | 1.90 | 1.28-2.82 |

Supplementary Table E4: Data for the FAB constructed variable.

The FAB variable includes patients at time point D2, having either activin A or B above the reference maximum, compared with patients who had neither activin A or B above the reference maximum, or patients at time point D2, who had both activin A and B above the reference maximum vs patients who had neither activin A or B above the reference maximum. This includes the area under curve (AUC), the positive likelihood ratio (LR+), the relative risk (RR) and the odds ratio (OR).

| Either A or B above Ref Max |  | **AUC** | **95% CI** | ***P* value** | **LR+** | ***P* value** | **95% CI** | **RR** | **95% CI** | **OR** | **95% CI** |
| --- | --- | --- | --- | --- | --- | --- | --- | --- | --- | --- | --- |
| vs Neither above Ref Max | 90 days | 0.59 | 0.52-0.66 | 0.015 | 1.54 | 0.004 | 1.12-2.00 | 1.75 | 1.21-2.55 | 2.057 | 1.27-3.34 |
|  | 12 months | 0.60 | 0.54-0.66 | 0.003 | 1.65 | 0.00032 | 1.28-2.12 | 1.83 | 1.32-2.53 | 2.30 | 1.46-3.62 |
| Both A and B above Ref Max |  | **AUC** | **95% CI** | ***P* value** | **LR+** | ***P* value** | **95% CI** | **RR** | **95% CI** | **OR** | **95% CI** |
| vs Neither above Ref Max | 90 days | 0.64 | 0.57-0.72 | <0.0001 | 2.57 | <0.00001 | 1.82-3.62 | 2.66 | 1.85-3.83 | 3.95 | 2.31-6.75 |
|  | 12 months | 0.65 | 0.58-0.72 | <0.0001 | 2.78 | <0.00001 | 1.96-3.95 | 2.61 | 1.90-3.58 | 4.33 | 2.58-7.27 |

Supplementary Table E5: Detailed Stratification of FAB Scores

Stratified FAB Scores and odds ratios with 95% confidence intervals (for 12 months), with the number of patients per stratified group and the number of patients who died within the 90 days and at 12 months. *P-*values refer to the appropriate comparisons with the reference group (FAB Score of 0.0 to 0.1).

| FAB Score | Odds Ratio and 95% CI for death at 12 months | *P* value | Mean Survival and 95%CI | Total Patients | Died within 90 days | Survived to 90days | Died within 12 months | Survived to 12 months |
| --- | --- | --- | --- | --- | --- | --- | --- | --- |
| 0.0-0.1 | Reference | Reference | 355.6 (344.4-366.7) | 140 | 4 | 136 | 7 | 133 |
| 0.1-0.2 | 2.691 (1.059-6.834) | 0.04 | 332.2 (311.6-352.8) | 93 | 8 | 85 | 12 | 81 |
| 0.2-0.3 | 3.667 (1.365-9.846) | 0.01 | 310.6 (276.4-344.9) | 52 | 8 | 44 | 9 | 43 |
| 0.3-0.4 | 9.744 (4.039-23.507) | <0.000001 | 238.2 (189.2-287.3) | 43 | 15 | 28 | 17 | 26 |
| 0.4-0.5 | 11.622 (4.884-27.655) | <0.0000001 | 228.4 (177.9-278.9) | 44 | 16 | 28 | 19 | 25 |
| 0.5-0.6 | 20.860 (8.974-48.486) | <0.0000001 | 154.0 (101.5-206.4) | 35 | 19 | 16 | 24 | 11 |
| 0.6-0.7 | 23.555 (9.592-57.844) | <0.0000001 | 150.3 (79.4-221.2) | 23 | 14 | 9 | 15 | 8 |
| 0.7-0.8 | 26.549 (11.257-62.614) | <0.0000001 | 119.0 (61.8-176.2) | 28 | 18 | 10 | 21 | 7 |
| 0.8-0.9 | 29.775 (11.979-74.011) | <0.0000001 | 100.7 (33.1-168.3) | 18 | 13 | 5 | 14 | 4 |
| 0.9-1.0 | 53.854 (16.298-171.325) | <0.0000001 | 25.4 (0.0-59.2) | 5 | 4 | 1 | 5 | 0 |

Supplementary Table E6

Data for Diagnostic Group at each time point (D0, D2 and D7) for Follistatin, Activin A and Activin B, *=p<0.05, **=p<0.01, ***=p<0.001 and ****=p<0.0001. IQR = Interquartile range (25%-75%).

|  |  | Follistatin (ng/mL) D0 | Follistatin (ng/mL) D2 | Follistatin (ng/mL) D7 | Activin A (ng/mL) D0 | Activin A (ng/mL) D2 | Activin A (ng/mL) D7 | Activin B (ng/mL) D0 | Activin B (ng/mL) D2 | Activin B (ng/mL) D7 |
| --- | --- | --- | --- | --- | --- | --- | --- | --- | --- | --- |
| Reference | Mean±SEM | 12.61±0.38 (n=138) | 12.61±0.38 (n=138) | 12.61±0.38 (n=138) | 0.11±0.00 (n=138) | 0.11±0.00 (n=138) | 0.11±0.00 (n=138) | 0.07±0.00 (n=138) | 0.07±0.00 (n=138) | 0.07±0.00 (n=138) |
|  | Median | 12.07 | 12.07 | 12.07 | 0.11 | 0.11 | 0.11 | 0.067 | 0.067 | 0.067 |
|  | IQR | 5.6 | 5.6 | 5.6 | 0.053 | 0.053 | 0.053 | 0.04 | 0.04 | 0.04 |
| 1-PO Cardiovascular | Mean±SEM | 16.47±0.58 (n=107)**** | 15.84±0.51 (n=114)**** | 14.26±0.57 (n=71) | 0.37±0.04 (n=107)**** | 0.24±0.01 (n=114)**** | 0.28±0.02 (n=71)**** | 0.44±0.04 (n=107)**** | 0.22±0.02 (n=114)**** | 0.19±0.02 (n=71)**** |
|  | Median | 16.51 | 14.96 | 13.33 | 0.31 | 0.2 | 0.24 | 0.35 | 0.15 | 0.16 |
|  | IQR | 9.46 | 6.86 | 6.91 | 0.24 | 0.14 | 0.19 | 0.35 | 0.18 | 0.20 |
| 2-PO Gastro-intestinal | Mean±SEM | 16.46±1.81 (n=55) | 14.22±0.90 (n=52) | 12.58±1.14 (n=42) | 0.47±0.05 (n=55)**** | 0.32±0.03 (n=52)**** | 0.36±0.05 (n=42)**** | 0.27±0.04 (n=55)**** | 0.15±0.01 (n=52)**** | 0.13±0.01 (n=42)*** |
|  | Median | 13.22 | 13.57 | 11.39 | 0.33 | 0.22 | 0.27 | 0.18 | 0.12 | 0.099 |
|  | IQR | 6.27 | 6.04 | 4.79 | 0.41 | 0.32 | 0.29 | 0.19 | 0.14 | 0.11 |
| 3-NO Cardiovascular | Mean±SEM | 17.70±0.82 (n=72)**** | 17.97±0.94 (n=71)**** | 12.98±1.01 (n=39) | 0.53±0.14 (n=72)**** | 0.36±0.06 (n=71)**** | 0.39±0.14 (n=39)**** | 0.30±0.05 (n=72)**** | 0.24±0.03 (n=71)**** | 0.21±0.06 (n=39)**** |
|  | Median | 16.63 | 15.81 | 11.87 | 0.24 | 0.23 | 0.2 | 0.18 | 0.15 | 0.12 |
|  | IQR | 7.33 | 8.85 | 4.29 | 0.28 | 0.19 | 0.2 | 0.17 | 0.19 | 0.13 |
| 4-NO Sepsis | Mean±SEM | 16.34±1.13 (n=25)* | 16.20±1.61 (n=26) | 12.55±0.99 (n=21) | 0.52±0.08 (n=25)**** | 0.44±0.12 (n=26)**** | 0.35±0.07 (n=21)**** | 0.38±0.07 (n=25)**** | 0.34±0.08 (n=26)**** | 0.15±0.03 (n=21)** |
|  | Median | 14.97 | 14.25 | 11.63 | 0.38 | 0.31 | 0.25 | 0.26 | 0.15 | 0.098 |
|  | IQR | 6.55 | 6.95 | 6.57 | 0.47 | 0.35 | 0.16 | 0.19 | 0.28 | 0.11 |
| 5-NO Gastrointestinal/Hematologic | Mean±SEM | 18.02±1.92 (n=23)** | 14.11±1.10 (n=22) | 12.32±1.06 (n=15) | 0.38±0.08 (n=23)**** | 0.40±0.04 (n=22)**** | 0.47±0.06 (n=15)**** | 0.34±0.11 (n=23)**** | 0.24±0.06 (n=22)**** | 0.15±0.02 (n=15)*** |
|  | Median | 15.56 | 13.13 | 12.32 | 0.25 | 0.41 | 0.45 | 0.16 | 0.17 | 0.12 |
|  | IQR | 7.3 | 6.27 | 6.4 | 0.19 | 0.3 | 0.33 | 0.16 | 0.16 | 0.11 |
| 6-NO Metabolic | Mean±SEM | 15.03±2.50 (n=21) | 12.71±1.05 (n=21) | 9.33±0.84 (n=11)**** | 0.26±0.07 (n=21)** | 0.20±0.03 (n=21)** | 0.23±0.03 (n=11)*** | 0.49±0.35 (n=21)* | 0.08±0.01 (n=21) | 0.08±0.01 (n=11) |
|  | Median | 13.35 | 12.52 | 8.54 | 0.16 | 0.17 | 0.28 | 0.11 | 0.05 | 0.086 |
|  | IQR | 9.36 | 8.855 | 5.06 | 0.15 | 0.095 | 0.16 | 0.097 | 0.07 | 0.066 |
| 7-NO/PO Neurologic | Mean±SEM | 12.22±0.85 (n=52) | 11.19±0.53 (n=51) | 8.29±0.65 (n=26) | 0.19±0.03 (n=52)** | 0.16±0.01 (n=51)** | 0.14±0.01 (n=26) | 0.12±0.02 (n=52) | 0.07±0.01 (n=51) | 0.06±0.01 (n=26) |
|  | Median | 11.12 | 11.01 | 7.78 | 0.14 | 0.14 | 0.14 | 0.077 | 0.054 | 0.046 |
|  | IQR | 5.575 | 5.53 | 3.83 | 0.07 | 0.1 | 0.075 | 0.086 | 0.056 | 0.044 |
| 8-NO Respiratory | Mean±SEM | 14.31±0.98 (n=81) | 11.34±0.53 (n=81) | 10.88±0.55 (n=55) | 0.29±0.03 (n=81)**** | 0.24±0.02 (n=81)**** | 0.26±0.02 (n=55)**** | 0.18±0.02 (n=81)**** | 0.13±0.02 (n=81)**** | 0.15±0.03 (n=55)*** |
|  | Median | 13.03 | 10.76 | 9.83 | 0.22 | 0.2 | 0.23 | 0.11 | 0.094 | 0.10 |
|  | IQR | 6.15 | 5.815 | 5.6 | 0.2 | 0.2 | 0.22 | 0.14 | 0.078 | 0.11 |
| 9-NO/PO Trauma | Mean±SEM | 10.59±0.70 (n=31) | 12.59±0.61(n=37) | 8.33±0.61 (n=28)**** | 0.16±0.01 (n=31) | 0.16±0.01 (n=37)* | 0.19±0.01 (n=28)*** | 0.11±0.01 (n=31) | 0.07±0.01 (n=37) | 0.06±0.01 (n=28) |
|  | Median | 10.52 | 12.08 | 7.56 | 0.13 | 0.15 | 0.18 | 0.092 | 0.062 | 0.057 |
|  | IQR | 5.42 | 5 | 4.12 | 0.08 | 0.11 | 0.12 | 0.102 | 0.058 | 0.0425 |
| 10-PO Respiratory/Gyne/Renal/Ortho | Mean±SEM | 11.90±0.87 (n=20) | 10.93±0.77 (n=20) | 12.93±0.92 (n=15) | 0.21±0.05 (n=20) | 0.21±0.04 (n=20)* | 0.24±0.04 (n=14)*** | 0.14±0.03 (n=20) | 0.12±0.02 (n=20) | 0.14±0.03 (n=15)* |
|  | Median | 11.98 | 9.68 | 13.51 | 0.14 | 0.15 | 0.18 | 0.1 | 0.093 | 0.1 |
|  | IQR | 7.19 | 5.87 | 3.75 | 0.17 | 0.12 | 0.22 | 0.083 | 0.086 | 0.072 |

Supplementary Table E7: ROC analysis for the FAB Score at 90 days and 12 months, organised by individual diagnostic group and combined

| ROC Curve (90 Days) | Area | Std Error | p-value | Lower Bound 95% CI | Upper Bound 95% CI |
| --- | --- | --- | --- | --- | --- |
| Group 1 | 0.760 | 0.073 | 0.001 | 0.617 | 0.903 |
| Group 2 | 0.927 | 0.036 | <0.0001 | 0.856 | 0.999 |
| Group 3 | 0.716 | 0.062 | 0.002 | 0.595 | 0.838 |
| Group 4 | 0.708 | 0.105 | 0.089 | 0.502 | 0.915 |
| Group 5 | 0.844 | 0.084 | 0.015 | 0.680 | 1.000 |
| Group 6 | 0.991 | 0.066 | 0.012 | 0.782 | 1.000 |
| Group 7 | 0.776 | 0.071 | 0.002 | 0.637 | 0.915 |
| Group 8 | 0.677 | 0.065 | 0.014 | 0.549 | 0.805 |
| Group 9 | 0.557 | 0.184 | 0.788 | 0.196 | 0.918 |
| Group 10 | 0.528 | 0.232 | 0.900 | 0.074 | 0.982 |
| Combined | 0.798 | 0.023 | <0.0001 | 0.753 | 0.842 |

| ROC Curve (12 Months) | Area | Std Error | p-value | Lower Bound 95% CI | Upper Bound 95% CI |
| --- | --- | --- | --- | --- | --- |
| Group 1 | 0.731 | 0.071 | 0.003 | 0.592 | 0.869 |
| Group 2 | 0.906 | 0.041 | <0.0001 | 0.825 | 0.986 |
| Group 3 | 0.808 | 0.053 | <0.0001 | 0.704 | 0.912 |
| Group 4 | 0.688 | 0.110 | 0.112 | 0.472 | 0.904 |
| Group 5 | 0.905 | 0.064 | 0.003 | 0.779 | 1.000 |
| Group 6 | 0.912 | 0.066 | 0.012 | 0.782 | 1.000 |
| Group 7 | 0.798 | 0.065 | <0.0001 | 0.671 | 0.925 |
| Group 8 | 0.714 | 0.060 | 0.002 | 0.596 | 0.832 |
| Group 9 | 0.539 | 0.132 | 0.824 | 0.280 | 0.798 |
| Group 10 | 0.500 | 0.144 | 1.000 | 0.218 | 0.782 |
| Combined | 0.804 | 0.022 | <0.0001 | 0.762 | 0.847 |

Receiver Operator Curve Analysis: Comparison of patients who lived and died at 90 days and 12 months. Area is the area under the curve and Std Error is the Standard Error.
